# How organizational research can avoid the pitfalls of a co-optation perspective: analyzing gender equality work in Austrian universities with organizational institutionalism

**DOI:** 10.1080/14616742.2016.1189672

**Published:** 2016-08-02

**Authors:** Angelika Striedinger

**Affiliations:** ^a^ Vienna University of Economics and Business, Wien, Austria

**Keywords:** Co-optation, organizational institutionalism, gender equality, universities

## Abstract

The concept of co-optation offers vocabulary to discuss how concerns and demands of feminist movements are transformed on their way to, and within, mainstream organizations and policymaking. However, applications of this concept can have problematic implications, failing to grasp the complexity of social change efforts and contributing to divisions, rather than alliances, between different groups that work and fight for gender equality. This article argues that conceptual tools from organizational institutionalism can help to avoid these pitfalls by capturing the ambivalence of organizational change initiatives, and allowing us to identify not only counterintentional effects, but also subtle and unexpected opportunities of organizational gender equality work. I illustrate my arguments with empirical examples from research on gender equality work in Austrian universities.

## Introduction

When we analyze gender equality work in organizations as a translation of feminist movement concerns into the societal mainstream, an obvious question is: How are these concerns and demands transformed, and what gets lost along the way? One way of investigating the implementation of feminist movement issues into the procedures and structures of mainstream organizations is through the concept of co-optation. Co-optation is defined as a process whereby “opponents adopt aspects of the *content* of a movement’s discourse, while subverting its *intent*” (Burke and Bernstein 2014, 831), and which “may have diluting, demobilizing, depoliticizing, and disempowering effects on the movement” (Coy 2013, 281). This resonates with the discomfort and dissatisfaction often experienced by feminists who observe how demands for a deeper change in gender relations and in the fundamental makeup of our societies morph into specific policy recommendations and programs, robbed of their radical edge and their transformative potential. The concept of co-optation directs a critical and reflective gaze at “the symbolic power of claims like ‘fairness,’ ‘rights,’ and ‘equality’ as they travel across ideologically diverse arenas” (Naples 2013, 149).

However, understanding organizational gender equality work as co-opted by other agendas and interests can also come with problematic implications (see Swan and Fox 2010; Eschle and Maiguashca 2014). In this article, I highlight two potential pitfalls of this concept. First, I argue that some accounts of co-optation fail to grasp the complexity of social change processes. Although they aim to counter simplistic unidirectional stories of how organizations and policy discourses take up gender equality as a goal, paradoxically their interpretations can end up simply inversing these accounts, rather than adding complexity. Second, I criticize approaches that see feminism as complicit in furthering managerial power and capitalist hegemony. Such accounts implicitly or explicitly contrast “good” with “bad” feminism, and thereby contribute to antagonisms, rather than alliances, between different groups that work and fight for gender equality.

This article is guided both by an interest in grasping the complexities of societal change, as well as by the normative stance that one of our tasks as feminist researchers is to make feminist theorizing useful for efforts toward transforming gender relations. To this end, I propose to embed analyses of co-optation in the wider theoretical framework of organizational institutionalism. This theoretical perspective offers analytical tools that take into account the ambivalence of initiatives that aim to change how organizations work, and allow us to identify not only possible counterintentional effects of these efforts, but also subtle and unexpected opportunities.

Both my criticism against the potential pitfalls of employing the co-optation concept, as well as my belief in organizational research as a way to avoid these pitfalls, grew out of empirical work on gender equality in Austrian universities in the context of a wider research project GenderChange in Academia (GENIA, http://genderchange-academia.eu)*.* On the one hand, the concept of co-optation contributed to our vigilance toward how universities portrayed their commitment to gender equality and how they integrated this issue in managerial governance procedures. On the other hand, it was simply not sufficient for grasping contradictions and inconsistencies in the organizations or understanding the perceptions and strategies of gender equality agents. This posed an interesting challenge: how could we build on critical analyses such as provided by the idea of co-optation, but also avoid fully dismissing the actions of equality officers in organizations as facilitating and legitimating other policy agendas? And in which instances can adaptation to other frameworks and procedures create opportunities for promoting feminist ideas? Organizational institutionalism (Greenwood et al. 2008) provided a helpful theoretical toolkit for tackling this challenge. It builds on a macro-perspective of understanding organizational procedures and practices as materializations of wider societal knowledge structures and belief systems (Thornton, Ocasio, and Lounsbury 2012) and simultaneously enables a focus on micro-level processes to analyze how these wider societal structures shape and are shaped by organizational actors (Lawrence, Suddaby, and Leca 2011).

Building on empirical examples of gender equality work in Austrian universities, I discuss how this theoretical perspective can help us understand efforts toward achieving organizational change, which can contribute to wider societal transformation. I first describe the context of the empirical research that inspired this article and then give a short overview of the basic ontological premises of organizational institutionalism. In the two sections that follow, I develop my analysis of the potentially problematic implications of applying the concept of co-optation to organizational gender equality work and outline conceptual tools based on organizational institutionalism.

## Research context: gender equality work in Austrian universities

GENIA investigated gender differences in academic career opportunities in the context of a changing Austrian higher education landscape. I was part of an interdisciplinary research team consisting of feminist academics with an interest in the gendered effects of higher education governance. Our approach toward changes in the higher education landscape was guided by a critical account of the transformation toward the “entrepreneurial university” (Clark 1998). In Austria, the focal point of this transformation was the reform of the University Act in 2002, which fundamentally redesigned the governance structures in the higher education sector following the ideas of New Public Management (Schimank 2000; Saravanamuthu and Filling 2004; Deem and Brehony 2005). Through a frame analysis of the policy discourse surrounding this reform, we started to develop an understanding of the complex interplay between the managerial and entrepreneurial shifts in the higher education discourse and efforts to promote gender equality (Kreissl et al. 2015). Although gender equality advocates could strategically build on these discursive shifts to strengthen the legitimacy of gender equality activities, “certain elements of gender critique are clearly incompatible with the dominant frames that structure the debate on higher education reforms” (Kreissl et al. 2015, 235).

Our findings resonated with critical analyses of gender equality work in academia, which observe that “[g]ender equality responsibility seems to be more promotion- and market-driven than to be a core issue of the decision-makers in terms of advancement of gender equality” (Bendl and Schmidt 2012, 491). These studies highlight two main concerns. The first concern has to do with the rhetorical justification of gender equality activities. By legitimizing the importance of equality work with reference to its contribution to business-related goals, the justification for equality policies and programs moves away from gender equality as an aim in itself, and toward goals of optimizing female human resources or improving the image of the organization (Barry, Chandler, and Berg 2007; Wetterer 2009; Ahmed 2012). The second point of concern is the adaptation of equality work to managerial auditing techniques (Power 1997), which relies on the extensive use of gender as a binary category. Within the context of managerial governance, the focus of equality work is put on formal, visible and quantifiable procedures; in essence, on “body counting” (Alvesson and Billing 2002). This mechanism can in effect lead to strengthening an essentialist view on what is *male* or *female*. It marginalizes feminist knowledge, which understands gender as a socially constructed category and as a dynamic relationship rather than static attribute (Alvesson and Billing 2002; Lind 2003; Auer and Welte 2007).

These analyses reflect what we could describe as the effects of co-optation of gender equality work. Several definitions of this concept highlight how ideas and terminology are included into policy agendas in such a way that the “concept itself is not rejected, but its initial meaning is transformed and used in the policy discourse for a different purpose than the original one” (Stratigaki 2004, 36). In their analysis of gender mainstreaming policies in Belgium, Meier and Celis (2011) highlight how gender mainstreaming became “a formalistic exercise whilst losing sight of its broader goal” (469). Stratigaki (2004) analyzes the EU policy discourse on gender mainstreaming, observing a transformation of meaning from policy objectives with feminist aims toward market-oriented objectives. According to such analyses, co-optation results in “the loss of [the] potential [of key concepts] for changing gender relations” (Stratigaki 2004, 32). What is more, “co-optation can lead to the creation of seemingly parallel institutions and organizations that nonetheless contradict the original meanings and intent as put forth by social movements” (Burke and Bernstein 2014, 833). For example, Fraser (2009) famously analyzes the overall trajectory and historical significance of second-wave feminism. She observes that feminism “as a general discursive construct” has “gone rogue” (114) and, rather than achieving “transformations of the deep structures of capitalist society” (107), this has in fact helped advance “women’s subordination in state-organized capitalism” (105).

Such accounts offer conceptual vocabulary to discuss a dilemma faced by feminist movements, namely that core concerns and goals, once they become part of mainstream of policymaking and organizations, are adapted to other priorities. In this process, the meanings of these concerns and goals are often emptied of their fundamental critique of gender relations, and resignified to instead strengthen other agendas, which in some cases could even be interpreted as counterproductive to the original intentions. For our analysis of gender equality in the context of managerial university reform in Austria, the concept of co-optation helped us make sense of how gender equality was addressed and justified in the policy discourse.

However, subsequent analysis of legal provisions and organizational gender equality activities strengthened our suspicion that, by focusing on co-optation, we missed out on important aspects of the story. Parallel to the outlined reforms, the government and universities substantially extended gender equality regulations, structures and activities. And, importantly, these developments cannot be interpreted as solely motivated by, and adapted to, the dominant higher education policy paradigms of competitiveness and managerial control. Several changes to the University Act established gender equality as a core principle in its own right, rather than a means to enhance competitiveness. The law now specifies universities’ duty to implement federal equality legislation, including a 50 percent women’s quota in all decision-making bodies in universities. Gender equality has to be integrated in managerial procedures of target definition and performance evaluation; however, in addition to this managerial dimension, gender equality work is still mainly characterized by advocacy and tackling discrimination. Furthermore, spaces for developing feminist knowledge continue to exist, since all universities have to integrate gender knowledge in their curricula. The University Act prescribes that Equal Treatment Commissions, consisting of academic and nonacademic staff and students, have to be involved in all decision-making procedures and every university has to have administrative structures specifically tasked with coordinating gender equality activities and gender research. While there are differences between universities in terms of financial and personnel resources dedicated to gender equality work and to gender research and in the status of organizational gender equality agents, as well as in the range of activities, our observations indicate overall improvements in these areas.

Our understanding of gender equality in universities became more refined when we started to interview organizational gender equality agents. One core empirical element of the GENIA project was case study research (Eisenhardt 1989; Yin 1994) in four Austrian universities. I analyzed archival data and conducted interviews with members of the university leadership and with organizational actors who were in charge of gender equality issues within these universities (see Table 1 for an overview of data sources). I approached these conversations following the guidelines of episodic interviews (Flick 2009, 185–190), using uniform prompts to introduce relevant topics and questions, but for the most part leaving it to the interviewees to talk about issues that were important to them. The interviews with gender equality agents often turned into long and intimate conversations, in which I gained insight into the struggles and dilemmas, as well as strategies and successes, that shaped their everyday experiences. These conversations presented a more complex and nuanced picture of gender equality work than suggested by my initial focus on co-optation. First, rather than only marginalizing feminist knowledge, managerial procedures could in fact provide the basis for developing projects that clearly retained a feminist character and provided a platform for criticizing the gendered-ness of organizational structures. And second, gender equality agents critically reflected upon possible counterintentional effects of their own work and developed subversive strategies for advancing a more holistic gender equality agenda.

**Table 1.** Data sources for case study research in the research project GENIA.

	Case study universities			
	A	B	C	D
Interviews (duration of 1–3 hours each)				
• Gender equality agents (gender equality office, equal treatment commission)	2	5	3	5
• University governance (rectorate, senate, works council)	3	4	4	3
• Administrative personnel (quality assurance, personnel administration)	2	2	–	3
Documents (between 1–200 pages each)				
• Gender equality (plan for women’s advancement, activity outline, report)	3	2	2	3
• Management (development plan, performance agreement, guidelines)	6	5	3	3

Several studies on organizational equality and diversity initiatives also express skepticism toward the concept of co-optation. One strand of this literature applies Foucauldian governmentality theory (Prügl 2011; Reeves 2012) to investigate gender mainstreaming and diversity management in organizations. Prügl (2011) concludes that, from this perspective, “the question of whether an engagement with the mainstream co-opts feminist struggles loses its meaning. There is no pure feminist knowledge outside governmentality untouched by the workings of power” (85). A second strand of this literature builds on the ambivalences of organizational change efforts. Swan and Fox (2010), for example, discuss the effects of professionalization and managerialization of diversity work in organizations. Rejecting “simplistic concepts” such as “good or bad diversity” (572), they highlight that it can be “difficult to tell whether particular strategies represent co-optation or resistance” (585) and that “these practices are not mutually exclusive or easy to pigeon-hole” (583). Instead, these are “temporal and dynamic processes” (586) with unstable and often unforeseeable effects. As I will show, organizational institutionalism follows a similarly differentiated approach as these perspectives and offers concrete conceptual tools to make sense of change efforts in organizations.

## Theoretical approach: organizational institutionalism

An organizational approach to studying efforts toward social change is particularly fruitful because wide areas of social life are coordinated in organized contexts (Scott and Davis 2007, 2–3). Who makes decisions, how are conflicts solved, how are rights and duties formulated and enforced and how are resources distributed? The way organizational structures and procedures answer these questions reflects the wider patterns of meaning and power relations that define our societies. Consequently, changes in very concrete and local organizational practices can be an indicator of, or an instigator toward, changes in the wider material and symbolic structures of our societies (Thornton, Ocasio, and Lounsbury 2012, 136). Organizational analysis therefore allows us to investigate wider societal change by analyzing the conditions for and character of organizational change, i.e. of transformations in the rules, norms and cognitive frames that shape organizational life (Scott 1995, 35).

Before outlining concepts for understanding organizational gender equality work and illustrating them with empirical examples, I first offer a short overview of the main ontological premises of organizational institutionalism.

Organizational institutionalism follows a social constructivist approach to studying organizational phenomena. What happens in organizations is not interpreted as a result of technical-rational deliberations, but instead as an instantiation of societal institutions. Institutions are defined as cognitive structures, as typifications of actions and actors (as conceptualized by Berger and Luckmann 1967, 72). The core feature of an institution is its continual reproduction by the members of a society based on its taken-for-grantedness. Institutions are *the way things are* and *the way things are done*. The justifications and explanations behind these institutions – *why* things are (done) the way they are (done) – is provided by what this strand of theory refers to as “institutional logics” (Friedland and Alford 1991; Thornton, Ocasio, and Lounsbury 2012). These logics, or *rationalities*, are equally socially constructed. What is portrayed as *rational* decision making in organizations, then, can be interpreted not as opposed to *values* such as equality, but as a representation of an institutional logic which is a socially constructed cognitive structure. Management tools, such as accountability systems and auditing, are as value laden and based on socially-constructed rationalities as other possible grounds of decision making, such as professional hierarchies, democratic requirements or principles of justice and equality.

The second important premise of organizational institutionalism is that it builds on a recursive understanding of the relationship between structure and agency (see Giddens 1984). This understanding is particularly prominent in the strand of institutional theorizing that investigates actors’ attempts to change their institutional environments, and calls such attempts “institutional work” (Lawrence and Suddaby 2006; Lawrence, Suddaby, and Leca 2009). What people in organizations do is based on organizational and societal structures; but it also simultaneously shapes, i.e. reproduces or changes, these structures. Accordingly, gender equality agents in organizations are necessarily embedded in the cognitive structures of their organizational frameworks and their activities are *based on* how these organizations work. By developing strategies, and persistently highlighting the importance and legitimacy of gender equality, they also *shape* how their organizations work. This understanding allows us to walk the fine line of embedded agency, without conveying the image of gender equality agents as either completely co-opted by other organizational priorities or as heroic individuals who are disembedded from the powerful forces of their institutional environment.

Building on these understandings of rationality and agency, the following two sections elaborate on empirical examples of gender equality work in universities to show how applications of the concept of co-optation can risk (1) presenting an oversimplified picture of organizational and societal processes, and (2) leading to conclusions that are harmful for feminist movements. I introduce theoretical concepts inspired by organizational institutionalism and propose to embed analyses of co-optation in this wider theoretical perspective in order to (1) improve our understanding of the complexity of the field and (2) produce research that strengthens feminist change initiatives.

## Understanding the complexity of organizational change initiatives

The first point of criticism against understanding organizational gender equality work as co-opted is that it presents an oversimplified picture of organizational processes and change efforts. I elaborate on this point by applying this lens to two examples of gender equality work in universities and then contrast this understanding with theoretical concepts inspired by organizational institutionalism.

The first example concerns gender equality work in the area of work–family responsibilities. Many Austrian universities integrated this topic in their performance agreements with the Higher Education Ministry as one of their target areas for improvements in gender equality, and this often included passing the audit program University and Family. The main thrust of this audit program was to enable reconciliation of professional career development and family responsibilities. This reflects what Stratigaki (2004) criticizes in the EU’s gender equality policy: while feminist movements framed the issue of family and care work as a critique of gender relations, demanding equal sharing of this work between women and men, the policy discourse has transformed this issue into a policy agenda that primarily aims at making sure family responsibilities do not interfere with women’s performance at the workplace. In this sense, if gender equality agents engage in initiatives under the umbrella of work–family responsibilities, their activities could be understood as co-opted by the market-oriented objective of increasing the performance of female human resources and void of the original feminist potential to transform gender relations.

In a second example of gender equality work, gender equality offices organized trainings aimed at reducing bias in personnel evaluation procedures. These trainings were part of the universities’ leadership development programs and they were explicitly framed as a way to increase the quality of personnel selection, rather than as a means to overcome inequality. The terms “gender,” “diversity” or “equality” were not even in the title of these trainings and did not feature prominently in their description; instead, the workshops were portrayed as part of the *quality assurance* agenda of the university. By applying the concept of co-optation, we could criticize this rhetorical framing as contributing to the marginalization of gender equality as an aim in itself, making the legitimacy of gender equality activities dependent on whether they contribute to other aims such as quality assurance and optimizing human resources (see Lind 2003; Barry, Chandler, and Berg 2007; Wetterer 2009).

These interpretations certainly help us develop a critical perspective on how feminist concepts and goals travel into organizational procedures, taking on new meanings and implications. However, they run the risk of simply inversing such accounts when they replace a story of gender equality success with a story of gender equality failure, thereby overlooking the complexity of efforts aimed at changing organizations and, consequently, societies.

As a first step toward a more differentiated picture, we need to understand that organizations, in our case universities, are not monolithic blocks governed by a homogenous market logic of how to make the most efficient use of the available human resources. To this end, a helpful concept provided by organizational institutionalism is institutional complexity (Greenwood et al. 2011), meaning that organizations are confronted with different prescriptions from multiple institutional logics and that these logics exist simultaneously in one and the same organizational context, even when they are partly contradictory. In Austrian academia, our research in the context of the GENIA project led us to identify four rationalities which shape how universities approach gender equality work (Striedinger et al. 2014, 18–19). Universities simultaneously offer different and overlapping justifications for their gender equality activities by arguing that: (1) in the spirit of “academic professionalism,” gender bias should be eliminated in order to come closer to the aim of objective and neutral evaluation and discovery; (2) following the logic of the “socially responsible university,” tackling the underrepresentation of women in academia is a moral obligation and a legally defined duty; (3) within the logic of the “entrepreneurial university,” gender equality work contributes to improving the image of the university and to the use of its human resources; and (4) according to the logic of the “managerial university,” gender equality regulations align academic decision making with managerially defined goals and increase the transparency of organizational procedures. As we can see, the concept of institutional complexity led us to a more fine-grained understanding of organizational rationalities toward gender equality.

Second, organizational contexts are not only places where multiple logics compete and coexist, but institutional logics can themselves be subject to transformation. This happens, for example, when a new logic is “translated” into an existing institutional field. This concept “refers to the notion that ideas change when they travel from one context to another” (Boxenbaum and Strandgaard Pedersen 2009, 185). In this process, “blending” (Thornton, Ocasio, and Lounsbury 2012, 162) means that dimensions of different logics are combined, resulting in new vocabularies and practices; “assimilation” (164) means that dimensions of one logic are incorporated into another logic and end up further supporting and justifying this logic. What is described here as assimilation is very similar to the concept of co-optation; however, an institutional logics perspective suggests that assimilation could also work the other way around. For example, elements of managerialism can be assimilated into (used for) purposes of gender equality work (for a similar suggestion based on frame analysis see Ferguson 2005, 33). Furthermore, the idea of blending indicates the possibility of a more equal merging of different logics.

Third, institutional work (Lawrence, Suddaby, and Leca 2009) can consist in efforts to translate, blend or assimilate institutional logics. Through strategy development, such as a “small wins approach” or a strategic use of different “language styles” (Meyerson and Scully 1995, 594–598), gender equality agents can make use of the institutional complexity of their organizational contexts and turn “an institutional wrinkle into a significant tear in the institutional fabric” (Reay, Golden-Biddle, and Germann 2006). Furthermore, even when the commitment of the organizational leadership to gender equality mainly serves as lip service to increase the legitimacy of the organization vis-à-vis demands for social justice and equality (see Meyer and Rowan 1977), gender equality agents can build on these rhetorical commitments (Hofbauer et al. 2015) and, through their implementation work, retranslate them toward more holistic interpretations of gender equality.

Returning to our two examples of gender equality activities in Austrian universities introduced above, we can apply these concepts to paint a more dynamic and fine-grained picture of gender equality work than if we solely relied on the notion of co-optation. When it comes to the issue of reconciliation of work and family responsibilities, one gender equality agent working in the university structure explained:

We can see that there is a lot of political will from the ministry and from the rectors to push this issue. And of course we’re not particularly delighted with how they frame this, just as a way to make it easier, to make it possible that women can be good mothers and good workers too. But then again, and I also talked about this with other gender equality officers from other universities: we try to reinterpret this more towards gender equality in care responsibilities, and we use it as an opportunity to question gender stereotypes. (Gender equality officer, university B)

As this statement shows, the transformation of feminist demands does not end with the “selective incorporation and partial recuperation” (Fraser 2009, 99) of these demands by other policy priorities. The politically prioritized University and Family audit program may have little in common with a feminist agenda of transforming gender relations, since it does not problematize the societal issue of the unequal distribution of care responsibilities between women and men. However, in the course of translating the political and organizational commitment to the reconciliation of work and family responsibilities into concrete gender equality activities and regulations, gender equality agents can reshape the character of this issue toward problematizing gender relations.

In the second example introduced above, the bias-sensitizing workshops, gender equality agents explained that the rhetorical framing of these trainings as a means toward increasing quality, rather than as a gender equality activity, was a purposeful rhetorical strategy:

We promote these workshops under the heading of “bias sensitizing.” With this terminology, we managed to get access to a completely different constituency than before. Before, we only reached the usual suspects; it was mainly people who were already aware of gender issues that participated in these kinds of activities … We are often confronted with the argument that gender equality is incompatible with, or stands in opposition to, quality or excellence. That’s why we build on the notion of quality, and on overcoming bias, in promoting our reflexivity training. (Gender equality officer, university D)

Through rhetorical framing, gender equality agents employ language styles that resonate with dominant institutional logics of academic professionalism – increasing the objectiveness of evaluation and assessments – and the managerial university – contributing to quality assurance systems. This way, their activities can reach a far wider audience than if their framing relied only on the rationale of social responsibility. Once these wider target groups are in these trainings, they also engage in discussions about gender equality as an aim in itself. Furthermore, through the framing of bias prevention, gender equality agents engage in logic blending between the logics of academic professionalism, the managerial university and the socially responsible university. Quality and equality, rather than being contradictory goals, are presented as mutually dependent on each other.

Overall, these examples of gender equality work in universities show that the organizational implementation of feminist concerns cannot be adequately understood as a black-and-white story of either comprehensive implementation or co-optation. It does not even seem appropriate to understand gender equality work as a balancing of scales between adaptation to organizational contexts versus remaining committed to a feminist agenda. Rather, gender equality agents strategically translate and blend institutional logics, building on the institutional complexity of their organizational contexts.

## Strengthening interaction between feminist movements and initiatives

My second point of criticism against analyses that understand gender equality work in organizations as co-opted is that they can end up promoting perspectives that are in effect harmful to feminist movements. First, they tend to portray feminism in a passive role, and mainstream organizations or policy discourse in an active role. This is particularly concerning in the context of critical analyses of gender relations. This is illustrated by the vocabulary employed, for example when Stratigaki (2004) writes about feminist policy goals being “used” (36), “hijacked” and “thwarted” (51), or when Fraser (2013) describes feminism as “capitalism’s handmaiden.” This language implicitly reproduces gendered imagery of active, dominant (male) capitalism with strategic agency, and passive, subordinate (female) feminism as a discursive pawn. Second, in promoting a dichotomy between “pure” and “co-opted” feminism, between the *real* feminist movement and a “strange shadowy version” of it (Fraser 2009, 114), some of these accounts build on a unified, homogenous idea of what feminism is – and what it is not. As Eschle and Maiguashca (2014) point out, “socialist feminism is romanticised, presented as an ideal type rather than as a concrete, internally complex, historically specific political project” (639). This can unintentionally fuel antagonism between different groups and individuals who work and fight for a transformation of gender relations, which in itself is counterproductive to feminist solidarity and a collectivist approach to changing society.

Through a co-optation lens, we could interpret several gender equality activities in universities as passive and *unfeminist*. For example, some gender equality officers we interviewed spent a lot of energy on implementing gender budgeting in their organizational procedures. Gender budgeting is a prime example of what has been criticized as a “body counting” approach to gender equality work (Alvesson and Billing 2002), relying on a binary and essentialist understanding of gender. Gender budgeting activities, which are highly work intensive, can be understood as diverting gender equality agents’ energies away from other activities, while at the same time strengthening – being a “handmaiden” to (Fraser 2013) – the extension of managerial control through detailed auditing practices. Another example of gender equality work in universities that is subject to feminist criticism (Morimoto and Zajicek 2014) is individual career support for female academics. Activities such as grant schemes, career trainings and mentoring programs for women comprised a large portion of gender equality programs in Austrian universities. Rather than tackling the gendered structures of the organization (Acker 1992), such activities aim at compensating for women’s disadvantage in male-dominated academia, to equip female academics with the necessary skills and qualifications to compete on a level playing field with their male colleagues. And instead of fostering collective feminist solidarity, these programs can be criticized as reinforcing the legitimacy of individual competition.

If we see gender equality agents who invest their energies in these kinds of activities as passively co-opted by other agendas and differentiate them from *real* feminism, it only takes a small step to “discipline contemporary strands of activism as insufficiently progressive” (Eschle and Maiguashca 2014, 642) and end up denouncing these individuals and their work. Such an approach overlooks that those who do this kind of work are capable of reflecting upon their own embeddedness and the possible counterintentional effects of their work. For example, gender equality agents critically evaluated their activities in the field of gender budgeting:

I have strong doubts about this … but the agreement with the rector is “Okay, we’ll try to do something useful with this.” But well, the problem is, with gender budgeting, we can only tackle things that are quantifiable. I think that’s counterproductive. In my opinion, what we need to do now is to focus on cultural intervention. Organization culture, work culture, perspectives on the world … And the question is whether we can then tackle these more subtle issues, the things that really matter. (Gender equality officer, university D)

Similarly, when it comes to individual career support for female academics, gender equality agents expressed skepticism about the character of these kinds of activities:

It’s always about making women adapt and mold themselves, so that they can persist in this academic system. There’s no understanding that there are certain structures in a culture, which should be tackled. The focus is still on making women “fit in.” … My understanding of gender equality is a different one, and I will continue to try to promote that, but it’s difficult, and I have to, well, the result is that I somehow have to adapt. (Gender equality officer, university B)

Overall, gender equality agents demonstrated a high level of reflexivity about their own role within the organization and about the historical development of organizational gender equality work in relation to the feminist movement as the following statement shows:

My position in the organization, it was achieved by women and for women. And now the position has a different status, a different meaning. On the other hand, we also gained a lot, you see, it’s strongly anchored, and strongly interlocked with organizational procedures. Because we’re more in the center, because our work just has stronger effects than before. With the original approach, from women for women, we could just about get resources from external women’s support programs and do small things, that’s it; gender mainstreaming, structure-related measures – forget it. Well, that’s the price you pay, right? I know that, and I can live with that. (Gender equality officer, university D)

As we can see in such statements, organizational gender equality work is characterized by a high degree of ambivalence. Rather than passively giving in to the pressures of organizational adaptation, gender equality agents maintained their beliefs about what feminism and gender equality meant to them.

Building on the idea of ambivalence in the context of heterogeneous institutional environments, Meyerson and Scully (1995) develop the concept of tempered radicals, defining them as “individuals who identify with and are committed to their organizations, and are also committed to a cause, community, or ideology that is fundamentally different from, and possibly at odds with the dominant culture of their organization” (586). This concept highlights the importance of multiple embeddedness (Meyerson and Tompkins 2007), i.e. embeddedness in contexts that are shaped by different institutional logics, for enabling reflective capacities. Not only does this allow gender equality agents to maintain alternative perspectives on organizational procedures, it also provides a context within which they can develop effective strategies for organizational change (for a similar argument based on the concept of femocrats see Miller and Razavi 1998). Consequently, when we observe that organizational gender equality activities are adapted to priorities and procedures of their organizational contexts and interpret them as passively co-opted, as distorted versions of what was once an emancipatory feminist agenda, we not only overlook the reflective capacity of gender equality agents but also contribute to divisions between organizational gender equality work and feminist activism, thereby damaging the very foundation that enables critical reflection and strategy development.

In addition to multiple embeddedness, organizational analysis, especially when it engages with social movement research, provides helpful concepts for analyzing how organizational gender equality work relates with feminist movement ideas. First, the idea of legacies highlights that “the embedding of social movement discourse and practice within conservative institutional frameworks holds out the possibility of continued social change, albeit in much less visible and dramatic ways” (Lounsbury 2001, 52). Social movements can continuously create “layers” of such legacies in mainstream organizations and institutions (Schneiberg and Lounsbury 2008, 665). This concept is compatible with positive assessments of co-optation, such as proposed by Ferguson (2005). She concludes her analysis of the co-optation of feminist arguments in the Bush Administration’s security discourse by celebrating co-optation as an indicator of “the gains we have made in framing women's rights as an important political issue” (572). Second, and related to this point, is the idea of sequencing (Schneiberg and Lounsbury 2008, 664–666). This concept proposes a processual understanding of institutionalization “as a sequence or interaction between contestation and mobilization around alternative visions of order, on the one hand, and more conventional institutional dynamics, on the other” (653). Sequencing can consist in a transformation “from ‘outsider’ to ‘insider’ movements” (665); in our case, from feminist movements outside of organizations toward activism and gender equality structures within organizations. Alternatively, and more accurately for our case, sequencing does not necessarily consist in the transformation of a movement. Instead, outsider movements can continue to exist parallel to insider movements, which can mutually develop each other and the institutional frameworks they target. I understand interaction and exchange between feminist movements and organizational gender equality agents as one of the most important preconditions for maintaining ambivalence as a fruitful basis toward developing strategies and effecting organizational and, consequently, societal change.

Overall, the insight into gender equality agents’ capacity to reflect on possible counterintentional effects of their own work shows that they are not passive pawns co-opted by other agendas. Instead, their reflective capacity provides a basis for the development of subversive strategies and for making use of unexpected opportunities. By taking into account the relevance of multiple embeddedness as a precondition for dealing with ambivalence and maintaining this reflective capacity, we can see the harmful effects of promoting a division between “good” and “bad” versions of feminism and gender equality, which some analyses that solely employ a co-optation lens imply. Instead, by thinking in terms of legacies of social movements, and sequencing or interaction between outsider and insider movements, we can avoid these problematic implications and arrive at more fruitful conceptualizations of organizational gender equality work.

## Conclusion

Building on examples of gender equality work in Austrian universities, the main argument of this article is that societal change, specifically feminist change through organizational gender equality work, cannot be understood as a simple story of fully implementing – or failing to implement – social movement concerns into organizational policies, structures and procedures. While the concept of co-optation can productively sharpen our perception of possible counterintentional effects of gender equality activities, it often fails to capture the reflective capacities of gender equality agents, as well as the opportunities that arise out of strategic adaptation to organizational rationalities.

In contrast, concepts inspired by organizational institutionalism, such as institutional complexity, translation and logic blending, allow us to analyze strategies of organizational gender equality work in heterogeneous institutional environments. Furthermore, this theoretical perspective enables us to appreciate legacies of social movements, to understand the sequentiality of social change and to consider that multiple embeddedness of gender equality agents can be maintained through interaction between outsider and insider movements. Consequently, approaching gender equality work from the perspective of organizational institutionalism can help us avoid potential pitfalls that come with the sole application of the co-optation lens, such as conveying passive imagery of feminism and contributing to antagonisms between those who work and fight for transforming gender relations. Rather than dismissing major elements of gender equality work as being co-opted, this approach leads to research results which can inform and enhance the strategic agency of those who invest their time and energy into achieving gender equality in organizations. As Ahmed (2007) writes:

[S]trategy means using the terms that would allow us to be heard, even when we might critique such terms. The hope of working within institutions is that we can separate our strategies from both intentions and outcomes: that we can “take on” such terms temporarily to challenge the distribution of power within organizations, but not be taken in by them (247).

My normative argument put forward in this article is that it is our task as feminist researchers to develop analyses that support, rather than hamper, this kind of strategy development. My theoretical and methodological argument is that we can do that by embedding accounts of co-optation in the wider theoretical perspective of organizational institutionalism.

## References

[CIT0001] Acker J. 1992 “Gendered Institutions. From Sex Roles to Gendered Institutions.” *Contemporary Sociology* 21 (5): 565–569. doi: 10.2307/2075528

[CIT0002] Ahmed S. 2007 “The Language of Diversity.” *Ethnic and Racial Studies* 30 (2): 235–256. doi: 10.1080/01419870601143927

[CIT0003] Ahmed S. 2012 *On Being Included: Racism and Diversity in Institutional Life*. London: Duke University Press.

[CIT0004] Alvesson M., and Billing Y. D. 2002 “Beyond Body-Counting: A Discussion of the Social Construction of Gender at Work.” In *Gender, Identity and the Culture of Organizations*, edited by Aaltio I. and Mills A. J., 72–91. London: Routledge.

[CIT0005] Auer M., and Welte H. 2007 “Social Positioning of Equal Opportunity Actors in Austria.” *Equal Opportunities International* 26 (8): 778–801. doi: 10.1108/02610150710836136

[CIT0006] Barry J., Chandler J., and Berg E. 2007 “Women’s Movements and New Public Management: Higher Education in Sweden and England.” *Public Administration* 85 (1): 103–122. doi: 10.1111/j.1467-9299.2007.00636.x

[CIT0007] Bendl R., and Schmidt A. 2012 “Revisiting Feminist Activism at Managerial Universities.” *Equality, Diversity and Inclusion: An International Journal* 31 (5–6): 484–505.

[CIT0008] Berger P., and Luckmann T. 1967 *The Social Construction of Reality. A Treatise in the Sociology of Knowledge*. London: Penguin Books.

[CIT0009] Boxenbaum E., and Strandgaard Pedersen J. 2009 “Scandinavian Institutionalism – A Case of Institutional Work.” In *Institutional Work: Actors and Agency in Institutional Studies of Organizations*, edited by Lawrence T. B., Suddaby R., and Leca B., 178–204. Cambridge: Cambridge University Press.

[CIT0010] Burke M. C., and Bernstein M. 2014 “How the Right Usurped the Queer Agenda: Frame Co-optation in Political Discourse.” *Sociological Forum* 29 (4): 830–850. doi: 10.1111/socf.12122

[CIT0011] Clark B. R. 1998 *Creating Entrepreneurial Universities: Organizational Pathways of Transformation*. New York: Pergamon Press.

[CIT0012] Coy P. G. 2013 “Co-Optation.” In *The Wiley-Blackwell Encyclopedia of Social and Political Movements*, edited by Snow D. A., Della Porta D., Klandermans B., and McAdam D., 280–281. Hoboken: John Wiley & Sons.

[CIT0013] Deem R., and Brehony K. J. 2005 “Management as Ideology: The Case of ‘New Managerialism’ in Higher Education.” *Oxford Review of Education* 31 (2): 217–235. doi: 10.1080/03054980500117827

[CIT0014] Eisenhardt K. M. 1989 “Building Theories from Case Study Research.” *The Academy of Management Review* 14 (4): 532–550.

[CIT0015] Eschle C., and Maiguashca B. 2014 “Reclaiming Feminist Futures: Co-Opted and Progressive Politics in a Neo-Liberal Age.” *Political Studies* 62: 634–651. doi: 10.1111/1467-9248.12046

[CIT0016] Ferguson M. L. 2005 “‘W’ Stands for Women: Feminism and Security Rhetoric in the Post-9/11 Bush Administration.” *Politics & Gender* 1 (1): 9–38.

[CIT0017] Flick U. 2009 *An Introduction to Qualitative Research*. 4th ed Los Angeles: Sage.

[CIT0018] Fraser N. 2009 “Feminism, Capitalism and the Cunning of History.” *New Left Review* 56: 97–117.

[CIT0019] Fraser N. 2013 “How Feminism Became Capitalism's Handmaiden – And How to Reclaim It.” *The Guardian*, October 14. http://www.theguardian.com/commentisfree/2013/oct/14/feminism-capitalist-handmaiden-neoliberal.

[CIT0020] Friedland R., and Alford R. R. 1991 “Bringing Society Back In: Symbols, Practices, and Institutional Contradictions.” In *The New Institutionalism in Organizational Analysis*, edited by Powell W. W., and DiMaggio P. J., 232–263. Chicago: University of Chicago Press.

[CIT0021] Giddens A. 1984 *The Constitution of Society: Outline of the Theory of Structuration*. Cambridge: Polity Press.

[CIT0022] Greenwood R., Oliver C., Suddaby R., and Sahlin K. 2008 *The SAGE Handbook of Organizational Institutionalism*. Los Angeles: Sage.

[CIT0023] Greenwood R., Raynard M., Kodeih F., Micelotta E. R., and Lounsbury M. 2011 “Institutional Complexity and Organizational Responses.” *The Academy of Management Annals* 5 (1): 317–371. doi: 10.1080/19416520.2011.590299

[CIT0024] Hofbauer J., Striedinger A., Kreissl K., and Sauer B. 2015 “Of Trump Cards and Game Moves: Positioning Gender Equality as an Element of Power Struggles in Universities.” In *Pierre Bourdieu, Organization, and Management*, edited by Tatli A., Özbilgin M., and Karatas-Ozkan M., 139–161. New York: Routledge.

[CIT0025] Kreissl K., Striedinger A., Sauer B., and Hofbauer J. 2015 “Will Gender Equality Ever Fit In? Contested Discursive Spaces of University Reform.” *Gender and Education* 27 (3): 221–238. doi: 10.1080/09540253.2015.1028903

[CIT0026] Lawrence T. B., and Suddaby R. 2006 “Institutions and Institutional Work.” In *Sage Handbook of Organization Studies*, 2nd ed., edited by Clegg S. R., Hardy C., Lawrence T. B., and Nord W. R., 215–254. London: Sage.

[CIT0027] Lawrence T., Suddaby R., and Leca B. 2009 *Institutional Work: Actors and Agency in Institutional Studies of Organization*. Cambridge: Cambridge University Press.

[CIT0028] Lawrence T., Suddaby R., and Leca B. 2011 “Institutional Work: Refocusing Institutional Studies of Organization.” *Journal of Management Inquiry* 20 (1): 52–58. doi: 10.1177/1056492610387222

[CIT0029] Lind I. 2003 “Gender Mainstreaming – neue Optionen für Wissenschaftlerinnen?” In *Gleichstellung in der Forschung. Organisationspraktiken und politische Strategien*, edited by Matthies H., Kuhlmann E., Oppen M., and Simon D., 173–187. Berlin: edition sigma.

[CIT0030] Lounsbury M. 2001 “Institutional Sources of Practice Variation: Staffing College and University Recycling Programs.” *Administrative Science Quarterly* 46 (1): 29–56. doi: 10.2307/2667124

[CIT0031] Meier P., and Celis K. 2011 “Sowing the Seeds of Its Own Failure: Implementing the Concept of Gender Mainstreaming.” *Social Politics: International Studies in Gender, State & Society* 18 (4): 469–489. doi: 10.1093/sp/jxr020

[CIT0032] Meyer J. W., and Rowan B. 1977 “Institutionalized Organizations: Formal Structure as Myth and Ceremony.” *American Journal of Sociology* 83 (2): 340–363. doi: 10.1086/226550

[CIT0033] Meyerson D. E., and Scully M. A. 1995 “Crossroads Tempered Radicalism and the Politics of Ambivalence and Change.” *Organization Science* 6 (5): 585–600. doi: 10.1287/orsc.6.5.585

[CIT0034] Meyerson D. E., and Tompkins M. 2007 “Tempered Radicals as Institutional Change Agents: The Case of Advancing Gender Equity at the University of Michigan.” *Harvard Journal of Law and Gender* 30: 303–322.

[CIT0035] Miller C., and Razavi S. 1998 *Missionaries and Mandarins: Feminist Engagement with Development Institutions*. London: Intermediate Technology Publications.

[CIT0036] Morimoto S. A., and Zajicek A. 2014 “Dismantling the ‘Master’s House’: Feminist Reflections on Institutional Transformation.” *Critical Sociology* 40 (1): 135–150. doi: 10.1177/0896920512460063

[CIT0037] Naples N. A. 2013 “"It's Not Fair!": Discursive Politics, Social Justice and Feminist Praxis SWS Feminist Lecture” *Gender & Society* 27 (2): 133–157. doi: 10.1177/0891243212472390

[CIT0038] Power M. 1997 *The Audit Society: Rituals of Verification*. Oxford: Oxford University Press.

[CIT0039] Prügl E. 2011 “Diversity Management and Gender Mainstreaming as Technologies of Government.” *Politics & Gender* 7 (1): 71–89. doi: 10.1017/S1743923X10000565

[CIT0040] Reay T., Golden-Biddle K., and Germann K. 2006 “Legitimizing a New Role: Small Wins and Microprocesses of Change.” *Academy of Management Journal* 49 (5): 977–998. doi: 10.5465/AMJ.2006.22798178

[CIT0041] Reeves A. 2012 “Feminist Knowledge and Emerging Governmentality in UN Peacekeeping.” *International Feminist Journal of Politics* 14 (3): 348–369. doi: 10.1080/14616742.2012.659853

[CIT0042] Saravanamuthu K., and Filling S. 2004 “A Critical Response to Managerialism in the Academy.” *Critical Perspectives on Accounting* 15 (4–5): 437–452. doi: 10.1016/S1045-2354(03)00037-6

[CIT0043] Schimank U. 2000 “Welche Chancen und Risiken können unterschiedliche Modelle erweiterter Universitätsautonomie für die Forschung und Lehre der Universität bringen?.” In *Universitäten im Wettbewerb. Zur Neustrukturierung österreichischer Universitäten*, edited by Titscher S., Winckler G., Biedermann H., Gatterbauer H., Laske S., Moser R., Strehl F., Wojda F., and Wulz H., 94–147. München: Rainer Hampp Verlag.

[CIT0044] Schneiberg M., and Lounsbury M. 2008 “Social Movements and Institutional Analysis.” In *The SAGE Handbook of Organizational Institutionalism*, edited by Greenwood R., Oliver C., Suddaby R., and Sahlin K., 648–670. Los Angeles: Sage.

[CIT0045] Scott W. R. 1995 *Institutions and Organizations*. Newbury Park: Sage.

[CIT0046] Scott W. R., and Davis G. F. 2007 *Organizations and Organizing: Rational, Natural and Open Systems Perspectives*. Upper Saddle River: Prentice Hall.

[CIT0047] Stratigaki M. 2004 “The Cooptation of Gender Concepts in EU Policies: The Case of ‘Reconciliation of Work and Family’.” *Social Politics: International Studies in Gender, State & Society* 11 (1): 30–56. doi: 10.1093/sp/jxh025

[CIT0048] Striedinger A., Kreissl K., Hofbauer J., and Sauer B. 2014 “Assimilation, Contradiction, Opposition: Gender Equality Logics and Institutional Complexity in Higher Education Reform.” Paper presented at the 30th EGOS Colloquium, Rotterdam, July 3–5.

[CIT0049] Swan E., and Fox S. 2010 “Playing the Game: Strategies of Resistance and Co-Optation in Diversity Work.” *Gender, Work and Organization* 17 (5): 567–589. doi: 10.1111/j.1468-0432.2010.00524.x

[CIT0050] Thornton P. H., Ocasio W., and Lounsbury M. 2012 *The Institutional Logics Perspective: A New Approach to Culture, Structure, and Process*. Oxford: Oxford University Press.

[CIT0051] Wetterer A. 2009 “Gleichstellungspolitik im Spannungsfeld unterschiedlicher Spielarten von Geschlechterwissen. Eine wissenssoziologische Rekonstruktion.” *GENDER Zeitschrift für Geschlecht, Kultur und Gesellschaft* 1 (2): 45–60.

[CIT0052] Yin R. K. 1994 *Case Study Research: Design and Methods*. 2nd ed Thousand Oaks: Sage.

